# Estimates of Marine Debris Accumulation on Beaches Are Strongly Affected by the Temporal Scale of Sampling

**DOI:** 10.1371/journal.pone.0083694

**Published:** 2013-12-18

**Authors:** Stephen D. A. Smith, Ana Markic

**Affiliations:** National Marine Science Centre, Southern Cross University, Coffs Harbour, New South Wales, Australia; University of Western Ontario, Canada

## Abstract

Marine debris is a global issue with impacts on marine organisms, ecological processes, aesthetics and economies. Consequently, there is increasing interest in quantifying the scale of the problem. Accumulation rates of debris on beaches have been advocated as a useful proxy for at-sea debris loads. However, here we show that past studies may have vastly underestimated the quantity of available debris because sampling was too infrequent. Our study of debris on a small beach in eastern Australia indicates that estimated daily accumulation rates decrease rapidly with increasing intervals between surveys, and the quantity of available debris is underestimated by 50% after only 3 days and by an order of magnitude after 1 month. As few past studies report sampling frequencies of less than a month, estimates of the scale of the marine debris problem need to be critically re-examined and scaled-up accordingly. These results reinforce similar, recent work advocating daily sampling as a standard approach for accurate quantification of available debris in coastal habitats. We outline an alternative approach whereby site-specific accumulation models are generated to correct bias when daily sampling is impractical.

## Introduction

Marine debris is a key threatening process for marine organisms, with reports of fatal interactions becoming all too frequent [1]–[3]. While the discovery of vast concentrations of debris in ocean gyres over the past 2 decades [4]–[6], mostly comprising plastics, has increased awareness of the issue, the low cost and broad utility of plastic continues to drive growth in its production, with 265 Million tonnes produced in 2010 [7] and a forecast of 300 Million tonnes by 2020 [8]. Plastics have been recorded from some of the remotest beaches on the planet [9] and it is consequently highly likely that debris-free beaches have been consigned to history. Gaining accurate information on how much debris is in the marine environment is a critical step in targeted management, and assessments of accumulation rates on beaches are often used to provide such estimates for coastal environments [10]–[12].

There has been increasing recognition that accumulation studies may underestimate available debris and that the scale of this underestimation is dependent on the interval between accumulation studies [13], [14]. In the majority of accumulation and trend assessment studies, sampling was conducted at a minimum frequency of monthly [15], [16]–[18]. However, a few studies have employed bi-weekly intervals [19], [20], a three-day interval [21] and daily intervals [13], [14], [22]. Unsurprisingly, the highest time-standardised accumulation rates result from daily surveys, but this timeframe is impractical for ongoing monitoring across numerous sites or for protracted periods.

Quite apart from the large range of methods applied to assessing marine debris densities on beaches [10], the lack of standardised approaches to accumulation studies makes it difficult to assess comparative debris loads at different sites (but note that substantial progress has recently been made through the development of recommended international protocols [23]). Further, translating debris loads on beaches into estimates of available debris in coastal waters is rendered almost impossible in most cases.

An understanding of overall debris dynamics is clearly needed to provide greater certainty about debris densities in coastal waters: one approach is to develop models based on empirical data [13]. Such models require metrics on the amount of debris arriving on a beach (loading rate [10]) as well as the relative importance of different removal pathways such as lateral drift [24], *in situ* burial [19], [25], Aeolian transport, re-suspension and wash-out [13], and cleaning [26]–[28]. However, given the range of additional factors that can affect accumulation rates (e.g. extreme weather events, smaller-scale morphology of beaches, proximity to population centres, visitation rates and the socio-economic background of visitors, and other factors operating over various temporal scales) [13], [14], [20], [24], [29]–[34], it is unlikely that one model will fit all situations [13]. Nevertheless, the development of such models will facilitate progressive understanding of accumulation rates and, importantly, how these are correlated with the availability of debris in coastal habitats [14].

In this study, we take the first step in developing a model of marine debris dynamics in subtropical eastern Australia by assessing the effect of temporal scale on the estimated daily loading rates for a small depositional beach. We do not attempt to relate the patterns to the many, specific factors affecting debris accumulation rates – rather, we simply present the model as the product of these factors. We then explore the implications of our findings with respect to bias in accumulation studies with intervals of up to 6 months.

## Methods

**Figure 1:**
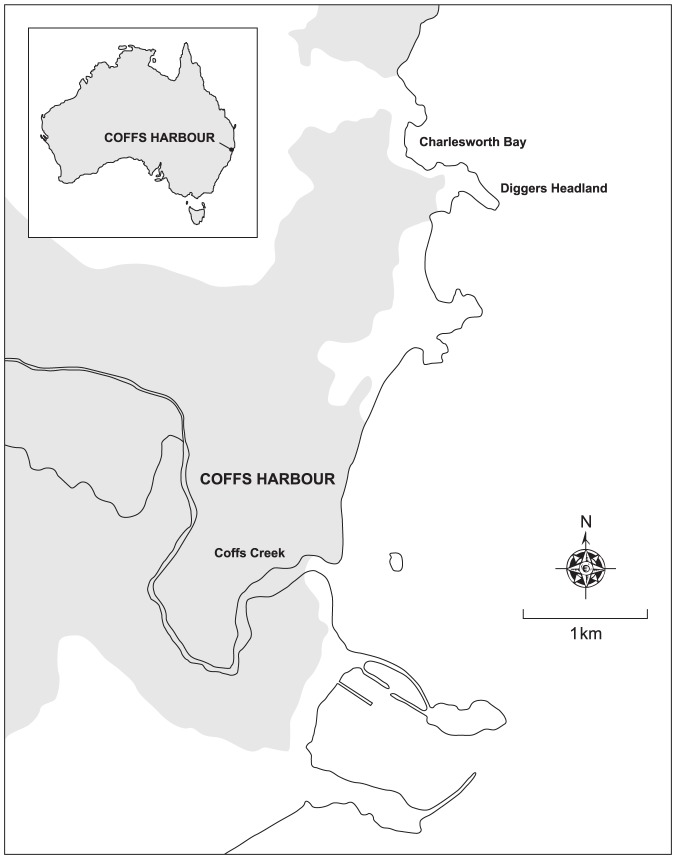
Map of the study area showing Charlesworth Bay and the extent of the urbanised area of Coffs Harbour (shaded).

Our study was conducted on a small (350-m long) beach, Charlesworth Bay (30.26692 S, 153.13975 E), immediately north of Coffs Harbour, the main population centre on this stretch of the NSW mid-north coast (Fig. 1). Charlesworth Bay is protected from the dominant south-easterly swell by Diggers Headland and its associated reefs. As a result, this beach experiences some of the lowest wave energy of those in the Coffs Harbour region [35], and is considered to be depositional. Classified as a reflective beach, it has a steep beach face with wave heights usually ≤0.5 m [35], [36]. Beach sediment consists of coarse sands and pebbles. Access to the beach is through a resort complex and visitation rates are substantially lower than for adjacent beaches with greater access (pers. obs.).

Sampling consisted of the removal of all items of macro-debris (≥5 mm) from the entire beach face. This involved searching from the waterline to the highest strandline, which was often within the vegetation line at the top of the beach. Each survey was completed over a single low-tide cycle. Only surface debris was removed and no attempt was made to exhume buried items unless they protruded through the beach surface.

Prior to commencing the accumulation study, we cleared the beach of all debris. Subsequently, over a 20-month period (July 2011 to March 2013), we conducted surveys of debris accumulation at intervals ranging from 1-165 days (Table 1). Given the considerable effort required to complete the surveys, some sampling periods were timed to coincide with larger community events (e.g. Clean Up Australia Day) or were carried out as a practical component in teaching (a 3^rd^ year undergraduate unit on marine pollution). In these latter situations, all removal was very carefully supervised to ensure that sampling intensity was the same as at other times. Converting debris loads to estimated daily accumulation rates, we modelled loss of debris from the beach by plotting estimated daily accumulation rate against the period of accumulation, and fitting the most parsimonious regression.

**Table 1:** Intervals used for accumulation studies and the number of items found in in each interval category (rounded to the nearest integer) (n =  the number of replicate surveys for that interval).

Interval (days)	n	Mean	Min.	Max.
1	7	772	540	928
4	1	1211	1211	1211
14	6	1506	825	2944
21	1	3762	3762	3762
28	2	4565	2080	7049
63	1	4571	4571	4571
84	1	1795	1795	1795
126	1	2360	2360	2360
165	1	5118	5118	5118

No permits were required for this study and the field work did not involve protected or endangered species.

## Results

The initial standing stock of debris at Charlesworth Bay was 4,044 items (0.24 items m^−2^) and we collected a further 42,684 items over the duration of the study. Plastic items contributed a total of 91.4% of the total debris. Within the plastics category, fragments of plastic (26.4% of total debris load) and monofilament fishing line (25.0% of total debris load), mostly from recreational fishing activities, predominated. Other plastic items included plastic bags (9.5% of total debris), food wrappers (7.4%), food containers (3.9%) and foamed plastic (styrofoam) (2.3%). The balance of the debris comprised items made from cloth (2.7%), metal (2.0%), rubber (1.6%), paper (0.8%), processed wood (0.8%), glass (0.6%) and “other” (0.1% - e.g. bricks and building materials). Of interest was the presence of 21 items that, based on date stamps (e.g. on plastic food containers), or information from the manufacturers, were 25–35 years old. These items primarily comprised plastic bottle tops, food containers and beer cans.

**Figure 2:**
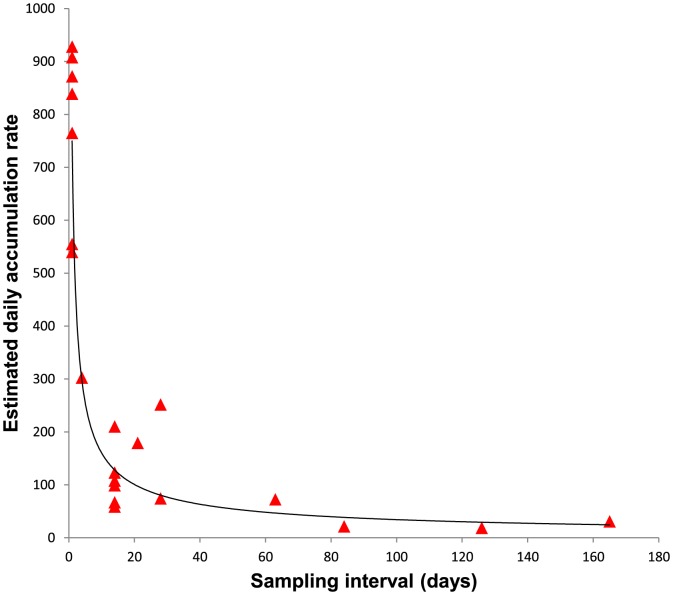
Plot of estimated daily accumulation rate of marine debris against interval between sample periods (days) for Charlesworth Bay. The regression is a power function with an *r* ^2^ of 0.872 and the following equation: *estimated daily accumulation (y)*  = 749.81*interval(x)* ^−0.67^.

The line-of-best-fit (*r* ^2^ = 0.872, *P*<0.001) for the plot of estimated daily accumulation rate against interval between samples was provided by a power curve (Fig. 2) with the following equation:
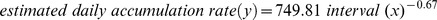



Thus, there was a rapid decline in estimated daily accumulation rate with increasing interval between sampling. Surveys conducted after an interval of only 3 days had daily accumulation rates <50% of the mean rate calculated from daily sampling, and apparent loads decreased by an order of magnitude by 1 month (∼30 days).

As previous studies have suggested that variation in estimated loads is greatest at the shortest intervals [10], we calculated the coefficient of variation (CV) for intervals at which we had 2 or more observations (intervals of 1, 14 and 28 days). There was a clear trend of increasing variation with increasing interval: 1 day, CV = 21.0: 14 days, CV = 49.0; 28 days, CV = 94.4 (but this was based on only 2 replicates – Table 1).

## Discussion

While this study was conducted on a single small beach, the results support those from other studies that have assessed accumulation over a range of temporal scales [10], [13], [14] - once deposited, debris is rapidly lost from the surface of beaches. Based on the few studies presenting comparable data, the rate-of-loss is surprisingly consistent regardless of geographic location. Thus, Eriksson et al. [14] found accumulation rates at Macquarie Island in the subantarctic to be underestimated by an order of magnitude after a period of one month. Similarly, Ryan et al. [10] reported that daily accumulation rates on beaches in South Africa were 100–600% greater than estimates based on weekly sampling: the modelled value in our study was ∼360%. This concordance between studies is made more remarkable given the likelihood of very different values of parameters known to affect debris accumulation. The implications of these studies are profound - that most accumulation studies vastly under-estimate the abundance of marine debris in coastal habitats and thus the scale of the problem is much greater than initially thought.

Although this study had the sole objective of generating an accumulation-sampling interval model, it is nevertheless instructive to examine the likely pathways through which debris is lost from the system. Many of these have been documented from previous beach debris work and it is highly likely that there is considerable interaction between most mechanisms.

Tidal inundation is a primary mechanism not only for transport of debris onto a beach [14] but also for removing it from the beach [21]. Different studies have found correlations between debris loads and the strength and direction of wind [14], [17], [19], which also affects the distribution of debris at the scale of the beach, including its burial. Extreme weather events (storms) can have a major impact through intensification of wind and wave action and through run-off into adjacent waterways. Intense storms may also introduce debris from adjacent subtidal habitats and this is likely to have been a principal source of the fishing monofilament that comprised 25% of the total debris load in this study. Monofilament does not float and most of the 11,660 pieces found during this study were entangled around kelp or other types of detached sessile benthos. This reflects the fact that monofilament is by far the most common type of debris found on local reefs, with the majority resulting from recreational fishing activities [37].

Burial is thought to be a major sink for debris on many beaches [38], although this is mediated by the size of the debris items relative to beach grain size [19]. The importance of this loss mechanism is emphasised by Kusui and Noda [25] who found that the average weight ratio of buried to stranded debris was 0.65 on beaches in Russia and Japan. Mechanical degradation is likely to be an important mechanism on beaches where abrasion processes are high. However, unless plastic debris arriving on a beach is already made brittle by photodegradation [22], it is unlikely to be a major contributor to short-term loss. Finally, with increasing public awareness about marine debris, and concerns for the health and aesthetics of beaches and marine environments [26], [39], [40], removal by visitors may be an important loss mechanism at popular beaches [41]. Clearly, the scale of removal will vary from place to place, and is unlikely to have contributed greatly to the observations in this study (on a beach that has low visitation rates), and would have been absent at Macquarie Island [14].

Whilst we have primarily focused on parallels with other studies, there are many additional site- and region-specific factors that are likely to result in different findings if a study such as this was conducted elsewhere. For example, beach width, slope, small-scale topographical features, proximity to debris sources (e.g. waterways or urban areas), and usage rates, all contribute to rates of debris retention and loss [11], [13], [34]. In addition, broad oceanographic patterns [20], [42], [43] have been demonstrated to influence accumulation rates in different geographic regions. Very sheltered beaches in the tropics may have the added factor of biogenic habitat (mangrove vegetation) that can trap and concentrate debris [44]–[46].

The necessity of daily sampling to gain a realistic estimate of loading rate for specific beaches poses a number of substantial challenges. Firstly, the sheer effort required to clean even a small beach, such as Charlesworth Bay, is considerable and thus incurs high time-costs. It is thus impractical to do this over a long-term period unless a large pool of volunteers is available. Indeed, the utility of volunteers has been widely recognised and they are, increasingly, being successfully engaged to deal with burgeoning worldwide debris loads [23], [41], [47]. However, the novelty of removing debris from a beach is likely to wear off, even for the most committed volunteers. Eriksson et al. [14] recognised this problem and suggested that, for example, instead of 12 samples at monthly intervals, accumulation studies should consider 12 consecutive days to provide more realistic estimates. However, this approach carries the inherent assumption that the 12 sampling days will be representative of the mean pattern of accumulation for the beach. In our study, we found that the coefficient of variation was lowest for daily accumulation rates with a progressive increase for intervals of 14 and 28 days. While this trend is opposite to that recorded by Ryan et al. [10], it was based on relatively few observations at the longest interval. Clearly, better estimates of temporal variation in daily accumulation rates are required before clear protocols can be recommended.

We suggest that there may be an alternative approach to repeated sampling over consecutive days – sampling at a range of intervals and constructing site-specific accumulation models as presented here. This would allow correction of future assessments of loading rates for sampling conducted at a variety of intervals: new data points can also be used to further refine the model. This is appealing as it not only provides models at a local scale, but also allows for less rigid sampling agendas in a habitat that can be difficult to work. However, to avoid bias with this approach, it is important that sampling is not simply opportunistic, or conducted in response to specific events (e.g. good weather, availability of volunteers): program planning should include appropriate *a priori* randomisation of sampling periods over the duration of the survey.

Despite the efforts of a number of research teams in the past [13], [19], it is clear that we still have a long way to go to generate realistic models for the dynamics of marine debris on beaches, let alone in less accessible habitats. This study helps to fill one of the gaps by providing a model for rates of accumulation and loss on an ocean beach. A key challenge remains not only to allocate the “lost” debris to the various possible pathways, but also to differentiate between, and quantify, the input sources which include: “new” items arriving by floating; items sourced from adjacent subtidal habitats; items delivered by wind and by runoff from adjacent terrestrial areas; and items retained within the system through a cyclical process of burial, exhumation and further transportation. The potentially extended temporal scale of the latter process is illustrated by the old items we found that had clearly been recently exhumed from adjacent beaches through coastal erosion.

This study provides strong evidence for rapid loss of debris from beaches following stranding, which has obvious implications for the interpretation of past accumulation studies. Thus, given that few studies have used sampling intervals of <1 month, and with the assumption that our model is more generally applicable, the scale of the marine debris problem in coastal waters may have been underestimated by at least an order of magnitude. This conclusion, which is supported by other recent studies [14], highlights the need for concomitant scaling up of measures to manage and mitigate the problem.
